# Distribution of virulence genes and their association with antimicrobial resistance among uropathogenic *Escherichia coli* isolates from Iranian patients

**DOI:** 10.1186/s12879-018-3467-0

**Published:** 2018-11-15

**Authors:** Yalda Malekzadegan, Reza Khashei, Hadi Sedigh Ebrahim-Saraie, Zahra Jahanabadi

**Affiliations:** 1 Department of Bacteriology and Virology, School of Medicine, Shiraz University of Medical Sciences, Shiraz, Iran; 2 Department of Urology, School of Medicine, Shiraz University of Medical Sciences, Shiraz, Iran

**Keywords:** *Escherichia coli*, Urinary tract infections, Antimicrobial resistance, Virulence genes

## Abstract

**Background:**

Urinary tract infections (UTIs) are one of the most frequent diseases encountered by humans worldwide. The presence of multidrug-resistant (MDR) uropathogenic *Escherichia coli* (UPEC) harboring several virulence factors, is a major risk factor for inpatients. We sought to investigate the rate of antibiotic resistance and virulence-associated genes among the UPECs isolated from an Iranian symptomatic population.

**Methods:**

A total of 126 isolates from inpatients with UTI from different wards were identified as UPEC using the conventional microbiological tests. After identification of UPECs, all the isolates were subjected to antimicrobial susceptibility test and polymerase chain reaction (PCR) to identify the presence of 9 putative virulence genes and their association with the clinical outcomes or antimicrobial resistance.

**Results:**

The data showed that the highest and the lowest resistance rates were observed against ampicillin (88.9%), and imipenem (0.8%), respectively. However, the frequency of resistance to ciprofloxacin was found to be 55.6%. High prevalence of MDR (77.8%) and extended-spectrum β-lactamase (ESBL) (54.8%) were substantial. PCR results revealed the frequency of virulence genes ranged from 0 to 99.2%. Among 9 evaluated genes, the frequency of 4 genes (*fimH*, *sfa*, *iutA*, and PAI marker) was > 50% among all the screened isolates. The *iutA*, *pap GII*, and *hlyA* genes were more detected in the urosepsis isolates with significantly different frequencies. The different combinations of virulence genes were characterized as urovirulence patterns. The isolates recovered from pyelonephritis, cystitis, and urosepsis cases revealed 27, 22, and 6 virulence patterns, respectively. A significant difference was determined between ESBL production with *pap GII*, *iutA*, and PAI marker genes.

**Conclusions:**

Our study highlighted the MDR UPEC with high heterogeneity of urovirulence genes. Considering the high rate of ciprofloxacin resistance, alternative drugs and monitoring of the susceptibility profile for UPECs are recommended.

**Electronic supplementary material:**

The online version of this article (10.1186/s12879-018-3467-0) contains supplementary material, which is available to authorized users.

## Background

Urinary tract infections (UTIs) are the most frequent human infections occurring to people of all ages, which cause morbidity and significant mortality globally [1, 2]. UTIs are mostly (70–90%) caused by the uropathogenic *Escherichia coli* (UPEC), one of the extraintestinal pathogenic *E. coli* pathotypes (ExPEC) [1, 3]. UPEC strains account for up to 90% of community-acquired UTIs and 50% of nosocomial UTIs [4]. UPEC strains usually carry a series of virulence markers, including adhesins, toxins and iron uptake systems (siderophores) that enable them to invade, colonize, and survive in the urinary tract, and prevent them from removal during urination [1, 4, 5]. Indeed, it is suggested that UPEC isolates usually harbor the largest number of pathogenicity-associated islands (PAIs) encoding a variety of virulence determinants involved in adhesion, invasion, and bacterial resistance to host defense and consequently influencing the pathogenicity of symptomatic or complicated UTIs [6, 7]. Initially, UPECs colonize the bladder and cause cystitis. Then, in an ascending manner would be able to move to the kidney, causing an acute pyelonephritis or disseminates to the blood leading to urosepsis [4, 6].

On the other hand, increasing antimicrobial resistance among UPEC isolates has been increasing dramatically and has turned out to be a serious health concern. The issue is mainly due to the emergence of multidrug-resistant (MDR) strains which contain genes encoding extended-spectrum β-lactamase (ESBLs) and resistance to sulfamethoxazole-trimethoprim (SXT) and fluoroquinolones [4]. UTIs caused by ESBL-producing UPEC strains are associated with prolonged hospitalization and hygiene cost [8]. The rate of MDR-UPEC strains in developed and developing countries is variable and in Iran as a developing country, has been estimated as 49.4% [9]. Given the increased resistance to the first line antimicrobial agents used in empiric therapy, UTI treatment in clinical practice has become somehow challenging. Indeed, the study of urovirulence genes and antimicrobial resistance can serve as a key element in developing new therapeutic targets. These factors can influence and be utilized as a useful marker to predict the clinical outcomes of UTIs caused by UPECs. There have been limited published epidemiologic studies on virulence genes and antimicrobial resistance among the UPECs isolated from symptomatic patients with UTI in Iran, hence, the present study aimed to evaluate the important characteristics of UPEC isolates, as well as investigate the correlation between the urovirulence genes and the type of clinical disease or antibiotic resistance from Shiraz, Iran.

## Methods

### Study population and bacterial isolates

A total of 126 non-repetitive *E. coli* isolates (one per patient) were obtained from inpatients who presented with symptomatic UTI at 3 teaching tertiary care hospitals (Nemazee, Faghihi and Dastgheib) in Shiraz, southwest of Iran, over a period ranging from April to August 2016. The study was approved by the Ethics Committee of Shiraz University of Medical Sciences (EC IR.SUMS.REC.1395.S77). The samples were collected as clean-catch midstream urine from the studied participants. UTI was defined as the presence of a positive urine culture (≥ 10^5^ colony-forming units [CFU]/mL) and pyuria (≥10^4^ leukocyte/mL of urine).

UPEC isolates were divided into three groups: 1) isolates associated with pyelonephritis (*n* = 73, 58%), 2) isolates associated with cystitis (*n* = 42, 33.3%), and 3) isolates related to urosepsis (*n* = 11, 8.7%). Fever, dysuria, urgent voiding, flank pain, nausea and vomiting are the characteristic symptoms of acute pyelonephritis. Cystitis was characterized by urinary frequency, internal dysuria and suprapubic or pelvic pain, whereas urosepsis was identified clinically by fever, tachycardia, tachypnea, and respiratory alkalosis. The isolation and identification *E. coli* strains were performed by standard microbiological and biochemical tests [10]. Confirmed *E. coli* isolates were kept frozen in tryptic soy broth (Merck Co., Germany) containing 20% glycerol (Merck KGaA, Germany) at − 70 °C until further experiments.

### Antimicrobial susceptibility testing

Antibiotic susceptibility of all isolates to ampicillin, ceftazidime, cefoxitin, gentamicin, amikacin, ciprofloxacin, sulfamethoxazole-trimethoprim (SXT or Co-trimoxazole), nalidixic acid, nitrofurantoin, and imipenem (Mast Co., UK) was carried out on Muller- Hinton agar (Oxoid Co., UK) using the disk diffusion method, as recommended by the Clinical and Laboratory Standards Institute (CLSI) [11]. *E. coli* ATCC 25922 was used as the quality control strain for antibacterial susceptibility testing. The isolates non-susceptible to ≥1 agent in ≥3 different antimicrobial categories were considered as MDR [12]. Moreover, the MIC (minimum inhibitory concentration) values of ciprofloxacin (as the most common antibiotic prescribed by clinicians in our region) were determined using the Epsilometer test (E-test). The test was performed with E-test strips (Liofilchem s.r.l., Roseto degli Abruzzi, Italy) as described by CLSI [11]. The isolates with MIC values ≤1, and ≥ 4 μg/mL were considered as susceptible, and resistant, respectively. Based on the antibiotic-susceptibility test results, the presence of resistance to both ciprofloxacin and nalidixic acid, indicates the isolates are marked as high-level quinolone-resistant bacteria, whereas nalidixic acid-resistant or intermediate isolates and ciprofloxacin-susceptible isolates are referred to as low level quinolone-resistant bacteria [13].

### Phenotypic detection of ESBL

Ceftazidime (as a third-generation cephalosporin) resistant isolates were selected for the ESBL test. ESBL-producing UPEC isolates were detected by the combined disk method using the ceftazidime-clavulanic acid (30/10 μg) disk. According to the CLSI guidelines, an increase of ≥5 mm in the diameter of the inhibition zones around disks containing clavulanic acid as compared to the inhibition zones around disks free of clavulanic acid indicated as ESBL producers [11]. *E. coli* ATCC 25922 and *Klebsiella pneumoniae* ATCC 700603 were used as negative and positive control strains, respectively.

### DNA extraction and virulence genotyping

Genomic DNA was extracted from all UPEC isolates using the Cinna-pure kit (CinnaGen Co., Iran) according to manufacturer^’^s instructions and subjected to polymerase chain reaction (PCR) after evaluating determining concentration and quality by measuring absorbance of A_260_ and A_280_ nm using a spectrophotometer and agarose gel electrophoresis, respectively. Isolated DNA was stored in Tris-EDTA buffer at − 20 °C until required for assays.

UPEC isolates were investigated for 9 virulence genes. The targeted genes and nucleotide sequences of the oligonucleotide primers used in this study were chosen as described elsewhere [14]. Simplex PCR was used to determine the presence of 9 virulence factors related to adhesion [*pap GI-III* alleles (P-fimbriae or pilus associated with pyelonephritis), *fimH* (type-1 fimbriae), *sfa* (S-fimbriae), *afa* (afimbrial adhesin)], toxins [*hlyA* (hemolysin)], siderophores [*iutA*], and PAI markers. Band sizes of the above-mentioned genes were 461, 190, 258, 508, 240, 559, 1177, 300, and 930 bp, respectively [14].

Amplification of DNA was performed using thermal cycler 5530 (Ependrof master, Germany), in a total volume of 25 μL, containing 3 μL DNA template, 2.5 μL PCR buffer (1X), 1 μL deoxyribonucleotide triphosphates solution (dNTPs, 200 μM), 1.5 μL MgCl_2_ (1.5 mM), 0.25 μL Taq DNA polymerase (1 Unit) and 1 μL of each specific primer (1 μM). The cycling conditions were set up as follows: 5 min at 94 °C as initial denaturation, 30 cycles of denaturation at 94 °C for 45 s, annealing (the temperature was depended on the primer sequence), extension at 72 °C for 1 min, and final extension at 72 °C for 10 min. All the reagents were obtained from Ampliqon Co., Denmark. Electrophoresis of amplicons was performed using 1.5% agarose gels, run in 1x Tris-acetate-EDTA buffer for 1 h in a horizontal electrophoresis system at 95 V. Gels were stained with safe stain load dye (CinnaGen Co., Iran) and visualized through UV transillumination.

### DNA sequence analysis

To confirm the accuracy of amplified gene of *afa* (two samples), the amplicons were submitted for sequencing (Bioneer Co., Munpyeongseoro, Daedeok-gu, Daejeon, South Korea) and the sequences were compared using online BLAST software (http://www.ncbi.nlm.nih.gov/BLAST/) (Additional file 1). For the rest of genes, *E. coli* ATCC 25922 was used as control strain.

### Data analysis

The Chi-square (χ ^2^) test was performed to analyze significant differences between the studied virulence genes with clinical outcomes or antimicrobial resistance, using SPSS (ver. 21.0; IBM Co., Armonk, NY, USA) software. The results of demographic and clinical manifestations were presented as descriptive statistics in terms of relative frequency. *P* value < 0.05 was considered to be significant.

## Results

## Subject characteristics

The recruited patients in our study were 50 females and 76 males aged from 1 to 100 years old with a mean age of 48.9 ± 28.8 years. There was no statistically significant difference in age and gender of subjects within the three studied disease groups. The recovered UPEC isolates from different wards were as follows: Intensive Care Unit or ICU (*n* = 76, 60.4%), Internal wards (*n* = 36, 28.6%), Surgery (*n* = 7, 5.6%), and Transplantation (*n* = 7, 5.6%). Moreover, the frequencies of cases in different wards were as follows: cystitis (ICU = 23, Internal ward = 12, Surgery = 2, Transplantation = 5), pyelonephritis (ICU = 44, Internal ward = 22, Surgery = 5, Transplantation = 2) and urosepsis (ICU = 9, Internal ward = 2, Surgery = 0, Transplantation = 0).

### Distribution of virulence genes

Frequency of the studied virulence genes among clinical groups is depicted in Table 1. Overall, 14.3% (18/126) of the UPEC isolates examined were positive for at least two of virulence markers. No significant difference was observed between virulence genes and isolates from different wards (data not shown). The frequency of only 4 genes (*fimH*, *sfa*, *iutA*, and PAI marker) was > 50% among all the isolates examined. The majority of virulence genes were determined in different proportions among the three clinical groups (Table 1). Among adhesins, the most prevalent gene in all groups was *fimH* (99.2%), followed by *sfa* (79.4%), and *pap GII* and *afa* were found 46%, equally. Neither *pap GI* nor *GIII* gene was detected among all of the clinical isolates. The *iutA*, *pap GII*, and *hlyA* genes were found essentially in urosepsis isolates with significantly different (*P* < 0.05) frequencies (Table 1). As shown in Table 1, among the clinical diseases, UPEC isolates recovered from urosepsis cases had the highest rate of designated genes. As for the distribution of the virulence genes, the isolates exhibited 35 distinct arrangements of virulence patterns, referred to as UPEC followed by an Arabic numeral (Table 2). The isolates recovered from pyelonephritis, cystitis, and urosepsis cases showed 27, 22, and 6 virulence patterns, respectively (Table 2). UPEC 1 was the most frequent pattern (16.7%), with the presence of *iutA*-*fimH*-PAI-*sfa*-*afa* virulence genes.

**Table 1:** Distribution of virulence genes among different clinical diseases

Genes	Cystitis No. (%)	Pyelonephritis No. (%)	Urosepsis No. (%)
*fimH*	41 (97.6)	73 (100)	11 (100)
PAI	36 (85.7)	61 (83.6)	11 (100)
*sfa*	33 (78.6)	58 (79.5)	9 (81.8)
*iutA* ^a^	24 (57.1)	52 (71.2)	10 (90.9)
*pap GII* ^b^	15 (35.7)	34 (46.6)	9 (81.8)
*afa*	19 (45.2)	36 (49.3)	3 (27.3)
*hlyA* ^b^	10 (23.8)	19 (26)	7 (63.6)

^a^The presence of this gene among urosepsis isolates was significantly higher than cystitis isolates (*P* < 0.05)

^b^The presence of this gene among urosepsis isolates was significantly higher than cystitis and pyelonephritis isolates (*P* < 0.05)

Abbreviations: *fimH* = type-1 fimbriae, *PAI* = pathogenicity-associated island, *sfa =* S-fimbriae, *iutA* = iron uptake transfer (ferric aerobactin receptor), *pap* = pilus associated with pyelonephritis, *afa* = afimbrial adhesion, *hlyA =* hemolysin

**Table 2:** Virulence patterns identified among UPEC isolates

Type No.	Virulence pattern	Total		Pyelonephritis		Cystitis		Urosepsis	
		No.	%	No.	%	No.	%	No.	%
UPEC1	*iutA-fimH-*PAI*-sfa-afa*	21	16.7	9	12.3	10	23.8	2	18.2
UPEC2	*papGII-iutA-fimH-*PAI*-hlyA-sfa-afa*	11	8.7	7	9.6	1	2.4	3	27.3
UPEC3	*papGII-iutA-fimH-*PAI*-hlyA-sfa*	11	8.7	4	5.5	4	9.5	3	27.3
UPEC4	*iutA-fimH-*PAI-*sfa*	9	7.1	6	8.2	3	7.1	0	0
UPEC5	*papGII-iutA-fimH-*PAI-*sfa-afa*	9	7.1	6	8.2	3	7.1	0	0
UPEC6	*fimH-sfa*	8	6.3	6	8.2	2	4.8	0	0
UPEC7	*papGII-iutA-fimH-*PAI*-sfa*	6	4.8	5	6.8	0	0	1	9.1
UPEC8	*fimH-*PAI	4	3.2	1	1.4	3	7.1	0	0
UPEC9	*iutA-fimH-*PAI	4	3.2	4	5.5	0	0	0	0
UPEC10	*fimH-*PAI*-sfa*	3	2.4	2	2.7	1	2.4	0	0
UPEC11	*papGII-iutA-fimH-*PAI	3	2.4	2	2.7	0	0	1	9.1
UPEC12	*iutA-fimH-*PAI*-afa*	3	2.4	2	2.7	1	2.4	0	0
UPEC13	*fimH-*PAI*-hlyA-sfa*	3	2.4	2	2.7	1	2.4	0	0
UPEC14	*papGII-fimH-*PAI*-sfa-afa*	3	2.4	1	1.4	2	4.8	0	0
UPEC15	*papGII-fimH*	2	1.6	2	2.7	0	0	0	0
UPEC16	*fimH-afa*	2	1.6	1	1.4	1	2.4	0	0
UPEC17	*papGII-fimH-sfa*	2	1.6	1	1.4	1	2.4	0	0
UPEC18	*iutA-fimH-sfa*	2	1.6	1	1.4	1	2.4	0	0
UPEC19	*papGII-fimH-*PAI*-sfa*	2	1.6	1	1.4	1	2.4	0	0
UPEC20	*papGII-fimH-*PAI*-hlyA-sfa*	2	1.6	2	2.7	0	0	0	0
UPEC21	*iutA-fimH-*PAI*-hlyA-sfa*	2	1.6	2	2.7	0	0	0	0
UPEC22	*iutA-fimH*	1	0.8	1	1.4	0	0	0	0
UPEC23	*fimH-hlyA*	1	0.8	0	0	1	2.4	0	0
UPEC24	*papGII-fimH-*PAI	1	0.8	0	0	1	2.4	0	0
UPEC25	*fimH-*PAI*-afa*	1	0.8	0	0	1	2.4	0	0
UPEC26	PAI*-sfa-afa*	1	0.8	0	0	1	2.4	0	0
UPEC27	*papGII-fimH-*PAI*-afa*	1	0.8	0	0	1	2.4	0	0
UPEC28	*iutA-fimH-*PAI*-hlyA*	1	0.8	1	1.4	0	0	0	0
UPEC29	*fimH-*PAI*-sfa-afa*	1	0.8	1	1.4	0	0	0	0
UPEC30	*papGII-iutA-fimH-*PAI*-afa*	1	0.8	1	1.4	0	0	0	0
UPEC31	*papGII-iutA-fimH-sfa-afa*	1	0.8	1	1.4	0	0	0	0
UPEC32	*papGII-fimH-*PAI*-hlyA-afa*	1	0.8	0	0	0	0	1	9.1
UPEC33	*fimH-*PAI*-hlyA-sfa-afa*	1	0.8	0	0	1	2.4	0	0
UPEC34	*papGII-iutA-fimH-*PAI*-hlyA-afa*	1	0.8	1	1.4	0	0	0	0
UPEC35	*iutA-fimH-*PAI*-hlyA-sfa-afa*	1	0.8	0	0	1	2.4	0	0

Abbreviations: *UPEC* = Uropathogenic *Escherichia coli*, *pap* = pilus associated with pyelonephritis, *iutA* = iron uptake transfer (ferric aerobactin receptor), *fimH* = type-1 fimbriae, *PAI* = pathogenicity-associated island, *hlyA =* hemolysin, *sfa =* S-fimbriae, *afa* = afimbrial adhesion

### Antimicrobial resistance among UPEC isolates

The highest resistance rate was observed against ampicillin (88.9%), followed by nalidixic acid (81%), sulfamethoxazole-trimethoprim (72.2%), ceftazidime (65.1%), ciprofloxacin (55.6%), cefoxitin (20.6%), gentamicin (19.8%), amikacin (7.9%), nitrofurantoin (4.8%), and imipenem (0.8%). The majority of isolates (*n* = 98, 77.8%) were MDR with predominant patterns for ampicillin, sulfamethoxazole-trimethoprim, nalidixic acid, ceftazidime, ciprofloxacin (15.1%), followed by ampicillin, sulfamethoxazole-trimethoprim, nalidixic acid, and ceftazidime with the frequency of 11.1%. Antibiotic susceptibility patterns were different depending on the place recovery of isolates. The frequencies of MDR isolates from ICU, Internal, Surgery, and Transplantation wards were 81.6, 69.4, 71.4, and 85.7%, respectively. Moreover, the isolates from urosepsis cases were more resistant than those recovered from cystitis and pyelonephiritis cases (81.8% vs. 78.6, and 76.7%, respectively, *P* > 0.05). Analysis of antibiotic resistance in terms of the gender and age of participants revealed no statistically significant differences among them (*P* = 0.82).

Relationship between the distribution of virulence genes and resistance to multiple drugs was also investigated. Among the studied genes, 100 and 78.6% of UPEC isolates harboring *fimH* and *sfa* were MDR, respectively. On the other hand, 60.7, 53.6, and 60.7% of the isolates carrying *iutA*, *pap GII*, and *hlyA* found to be susceptible to antimicrobial agents, respectively (data not shown). Further analysis revealed that the rate of ESBL-producing isolates was 54.8% (69/126). There was a significant correlation (*P* < 0.05) between ESBL-producing isolates and antibiotic resistance to all the antibiotics tested, except for amikacin, nitrofurantoin, and imipenem (Table 3). A significant difference was also observed between ESBL production and MDR positive isolates (97.1% ESBL producers vs. 54.4% non-ESBL producers, *P* < 0.001). Among the seven evaluated genes, a statistically significant difference was determined between ESBL production with *pap GII*, *iutA*, and PAI marker genes, and ESBL-negative isolates with *afa* gene (Table 4).

**Table 3:** Distribution of antibiotic resistant UPEC isolates according to ESBL production

Antibiotic	ESBL-negative	ESBL-positive	*P* value
	Resistant No. (%)	Resistant No. (%)	
Co-trimoxazole	36 (63.2)	55 (79.7)	0.039
Ampicillin	43 (75.4)	69 (100)	< 0.001
Nalidixic acid	35 (61.4)	67 (97.1)	< 0.001
Amikacin	2 (3.5)	8 (11.6)	0.095
Nitrofurantoin	2 (3.5)	4 (5.8)	0.54
Ceftazidime	13 (22.8)	69 (100)	< 0.001
Imipenem	0	1 (1.4)	0.36
Gentamicin	5 (8.8)	20 (29)	0.005
Ciprofloxacin	25 (43.9)	45 (65.2)	0.016
Cefoxitin	17 (29.8)	9 (13)	0.021

Abbreviations: *UPEC* = Uropathogenic *Escherichia coli*, *ESBL* = extended-spectrum β- lactamase

**Table 4:** Distribution of virulence genes among UPEC isolates according to ESBL production

Gene	ESBL-negative	ESBL-positive	*P* value
	Positive No. (%)		
*pap GII*	20 (35.1)	38 (55.1)	0.03
*iutA*	31 (54.4)	55 (79.7)	0.004
*fimH*	56 (98.2)	69 (100)	0.45
PAI	42 (73.7)	66 (95.7)	0.001
*hlyA*	15 (26.3)	21 (30.4)	0.69
*sfa*	42 (73.7)	58 (84.1)	0.19
*afa*	35 (61.4)	23 (33.3)	0.002

Abbreviations: *UPEC* = Uropathogenic *Escherichia coli*, *ESBL* = extended-spectrum β- lactamase, *pap* = pilus associated with pyelonephritis, *iutA* = iron uptake transfer (ferric aerobactin receptor), *fimH* = type-1 fimbriae, *PAI* = pathogenicity-associated island, *hlyA =* hemolysin, *sfa =* S-fimbriae, *afa* = afimbrial adhesin)

According to disk diffusion results, 60 (47.6%) isolates were high-level quinolone-resistant bacteria with ciprofloxacin MIC ≥6 μg/mL, while 42 (33.3%) isolates were identified as low level quinolone-resistant bacteria (MIC ≤1). In overall, the MIC range of all 70 ciprofloxacin-resistant isolates was between 6 and > 32 μg/mL, and both MIC_50_ and MIC_90_ were estimated > 32 μg/mL.

## Discussion

A better knowledge of the virulence markers of UPEC strains, especially in hospitalized patients allows the physicians to follow up the trend of pathogenicity of strains causing the urinary tract infections. The studied samples in the present investigation were originated from 126 inpatients with pyelonephritis, cystitis and urosepsis, which were evaluated for the presence of nine urovirulence genes and their corresponding antibiotic susceptibility patterns.

Genes encoding adhesins are the most frequently occurring virulence factors in UPECs [15]. Fimbriae are important to establish the UTI and probably in the progression to urosepsis [16]. It is suggested that type 1 and P fimbriae are common among cystitis and pyelonephritis-associated UPEC strains, respectively [5]. As we expected, in our study almost all the isolates (99.2%) carried the *fimH* gene, encoding of the type 1 fimbriae, consistent with some previous reports [1, 5, 7, 17, 18]. Conversely, in a recent study from Mexico, the prevalence of *fimH* was reported 61.3% [15], which was in agreement with some other published data [19, 20]. Recently, in an investigation on 183 UPEC isolates [5], the *fimH* was found to be associated with cystitis cases, but in the current work no correlation was found with clinical manifestations. The *sfa* was the second most prevalent adhesion gene (79.4%) in our isolates, consistent with a study conducted in South Korea with frequency of 100% [1]. On the contrary, in some reports the prevalence of less than 50% [7, 21, 22] or even 0% [23] was cited for this gene. Although the exact role of S-fimbriae is not identified; however, the dissemination of bacterium within the host tissue is suggested for this adhesin [1]. In the present study, the frequency of *pap GII* and *afa* were found to be 46%. It was reported that class II *pap G* allele is related to pyelonephritis cases, while *pap GIII* is primarily associated with UTIs in dogs and cats [18], which is in agreement with our findings. In two studies from South Korea and China [7, 18], no *pap GI* gene was identified in their isolates, similar to our findings, either. Indeed, it has been suggested that there is a possibility of mutation at the level of a specific gene, resulting in the absence of the corresponding gene in PCR method [19]. The role of *afa* afimbrial adhesin is mentioned in the development of chronic nephritis [19]. Our findings revealed that 49.3% of isolates from pyelonephritis cases were *afa* PCR-positive which is higher than those reported by other investigators [1, 15, 17–19, 22–24]. This discrepancy could be due to differences in type disease (symptomatic or asymptomatic bacteriuria) or geographic region.

The second most common gene in this study was found to be PAI marker (85.7%). The determinants such as toxins, siderophores, and protectins are encoded on UPEC PAIs [15]. This frequency was higher than those previously reported for UPEC isolates [15, 23, 24], but lower than that reported in other studies from Iran [25–27].

The *iutA* as a siderophore marker donates the potency of resistance against serum killing to the UPEC strains, thereby enabling them to persist in body fluids such as the blood [24]. The attributed characteristic is important for the pathogenesis of isolates causing urosepsis. As indicated by present findings, 90.9% of UPEC were carrying the *iutA* gene, suggesting the isolates are invasive and a significant association between this gene and clinical groups. According to the obtained data, the frequency in the current study was higher than those previously reported [7, 15], indicating the genes codifying siderophore vary depending on geographic areas and hosts [15]. However, of the three clinical complications, isolates recovered from urosepsis cases had the highest frequency among the studied genes.

The *hlyA* toxin is involved in tissue damage and impairment of local immune responses [19]. There was a significant association between *hlyA* gene and urosepsis isolates, which is consistent with invasive nature of UPECs isolated from urosepsis cases. Our results (28.6%) are in agreement with those found by other studies [7, 19, 28].

On the other hand, in the present investigation, 35 patterns of combinations of the urovirulence markers were characterized. The UPEC 1 pattern with *iutA*-*fimH*-PAI-*sfa*-*afa* template was the most frequently present. According to different geographic regions and disease status, different patterns of combinations of the virulence genes and antimicrobial resistance phenotypes have been reported in previous studies [7, 15, 23].

According to our data, all UPECs contained at least two virulence genes. This is in contrast to Oliveira et al. [24], who reported 90% UPECs showed at least one of the eight virulence genes. It was shown in a study [19], that UPECs isolated from hospitalized patients offered a great diversity of gene associations, in agreement with our data, indicating heterogeneity in the distribution of virulence genes among UPEC strains in different regions [24].

Increased antibiotic resistance, particularly for third-generation cephalosporins and fluoroquinolones among UPEC isolates has created challenges in clinical practice [29]. Majority of our isolates were remarkably resistant to the most of the tested antibiotics, with 77.8% of strains showing multi-drug resistance, making them the causative agent of an important health problem in our area, in agreement with previous works from different regions [15, 20, 25, 30]. As empirical antimicrobial therapy is usually the first conventional treatment for UTIs, awareness of the local epidemiological data for an efficient therapy is necessary and useful [31]. According to our investigation, 88.9, 81, 72.2, and 55.6% isolates were resistant to ampicillin, nalidixic acid, sulfamethoxazole-trimethoprim, and ciprofloxacin, respectively, the therapeutic agents used as the first-line empirical treatments for UTIs [24, 32]. Ciprofloxacin is the most common fluroquinolone used to treat UTIs. However, due to its overuse in the last decade, the resistance rate of UPECs to that antibiotic has markedly increased [33]. In comparison to other studies from different countries [18, 20, 24, 28], our rate (55.6%) is high, but it is consistent with another investigation from Iran with frequency of 61.3% [30]. Our findings regarding the MIC study of ciprofloxacin showed that the MIC_50_ and MIC_90_ ranges are higher than their corresponding maximum values in the E-test strip (32 μg/mL). The MIC values of clinical isolates vary based on geographic area and time. In a study from Algeria [28], ciprofloxacin MIC range was mentioned between 0.5 to > 128 μg/mL. It seems that in a clinical setting, we cannot surmount this level of resistance even by using the manifold dosages of MIC_50_ and MIC_90_ values. One explanation for our observed high rate in our region could be the wide use of antibiotics for bacterial infections, for example prescription of ciprofloxacin by clinicians in the first visit of patients with uncomplicated UTIs.

Similar to the other studies from Iran and other countries [1, 18, 20, 34, 35], the majority of isolates in the present study were susceptible to imipenem (99.2%) and nitrofurantoin (95.2%). Because of the emergence of antibiotic resistance in Gram-negative rods, imipenem is not preferably included in the first line therapy of UTIs and is recommended as a choice agent for only ESBL-producers [28, 32]. Additionally, in spite of good activity of nitrofurantoin against UPEC isolates, but due to numerous side effects, its application is limited. Nevertheless, because of increasing resistance rate of UPEC strains to sulfamethoxazole-trimethoprim and quinolones, rational use of nitrofurantoin has been recommended again for the re-infection prophylaxis of recurrent non complicated UTIs [36, 37].

Appropriate diagnosis and treatment of UTIs caused by ESBL-producing phenotypes is important for the prevention of long-term clinical outcomes [8]. One of our major concerns is about the observed high rates of MDR among UPEC ESBL-producers (97.1%). The data from a multi-centric study revealed that the rates of UPEC ESBL-producers among Iranian isolates were 42, and 44% of isolates which were MDR [38]. In the present study, the rate of ESBL-positive isolates was 54.8%, which was similar to some other studies [18, 34]. In contrary, in three studies from Iran a range of 22.3–35.7% was observed for UPEC ESBL-producers [20, 39, 40]. Such discrepancies might be due to the differences in epidemiology of isolates or sample size of studies.

## Conclusion

In summary, our data point out the battery of multi-drug resistance and genetic heterogeneity among UPEC isolates from southwest of Iran. Moreover, it can be suggested that antibiotic resistance is associated with the isolates harboring certain urovirulence genes, such as *sfa* or the presence of *iutA*, *pap GII* and PAI marker in ESBL-producers. Taking into account the results, it seems that ciprofloxacin could not be used in empiric antibiotic treatment and the alternative drugs such as cefoxitin and for cystitis cases, nitrofurantoin might be choice options. Taken together, our findings support the importance of some urovirulence genes (e.g., *iutA*, *pap GII* and *hlyA*) as a marker for developing of symptomatic UTIs. To our knowledge, the present work is the first study in Iran that characterizes the several urovirulence genes in different disease groups and warrants further intensive studies.

## Additional file

Additional file 1: The sequencing results of the *afa* gene. (DOCX 485 kb)

## Abbreviations

*afa*: Afimbrial adhesion
ATCC: American type culture collection
CFU: Colony-forming units
CLSI: Clinical and laboratory standards institute
ESBL: Extended-spectrum β-lactamase
E-test: Epsilometer test
ExPEC: Extraintestinal pathogenic *E. coli*
*hlyA*: Hemolysin
MDR: Multidrug resistant
MIC: Minimum inhibitory concentration
PAIs: Pathogenicity-associated islands
*pap*: P-fimbriae or pilus associated with pyelonephritis
PCR: Polymerase chain reaction
*sfa*: S-fimbriae
SXT: Sulfamethoxazole-trimethoprim
UPEC: Uropathogenic *Escherichia coli*
UTI: Urinary tract infection
