# Supplementary material for: Distribution of virulence genes and their association with antimicrobial resistance among uropathogenic Escherichia coli isolates from Iranian patients
Source: BMC Infect Dis. 2018 Nov 15;18:572. doi: 10.1186/s12879-018-3467-0 (PMC6238375; doi:10.1186/s12879-018-3467-0)

**Additional file 1:** The Sequencing result of the *afa* gene (Sample 1)

**Nucleotide Sequence:**

ATCGCCGTCAGGTCAGCACAGGCCTCCTCCGCCACGGAGGCACCACGACCCTCTCCGGATGCACGGAACAACGCCGGATATTCCTCAATCCTGACCCCGTAACGCGCCAGCATCTCACGGGTCAGACAGGTCTTCAGATAAGGCCTGCCCTCCGCGTCCCTCTCCGCGTGAAAGGCCATCTCCTGTGAATCCACACGGGAACCATTCAGGATAATGTCAACCGGATAAATGCCGGGTAACTGCCCACCTTCCTCAAACAGGGTCAGGTCCACCCCCTTACCGCCACCTTTCAGCATGGCCGCATCAAAGGAGTAGGTGCGCGCAATACCACTCTCCGCACGAAACATCACACAGGCCACCATCACAGCCAGCGCCAGCCCTGTCACCCCCGTTTTCATCCGCCCTGAAGAAGTATCACGCATCTGTCAGCCCGCCATTTACCCTGTTATCACCTTATGTATTCAACCCTTCAGCTCTGCCTCAAACTGCTTACTGGTCCCGCCGTAATCCGTTATCACCTTCCACTGAACCTTACCCGATGCCCCCGCAGGCAGCGGATATTCACGGGAGGAAAACGGCGCAATATATTCACGCTCCTTCACTTCCTTACCCCCCACCGTCAGCGTGGACAGGTTGATGTAAAACGGCGTCGGGTTAACCCCCTTCAGCCTGTTGCCGGCCCTCTGCCACTCCACCTTGCCGGCCACATCATCCGGTCGCCCCTTCACCGC

**Results of Nucleotide BLAST through https://blast.ncbi.nlm.nih.gov/Blast.cgi**


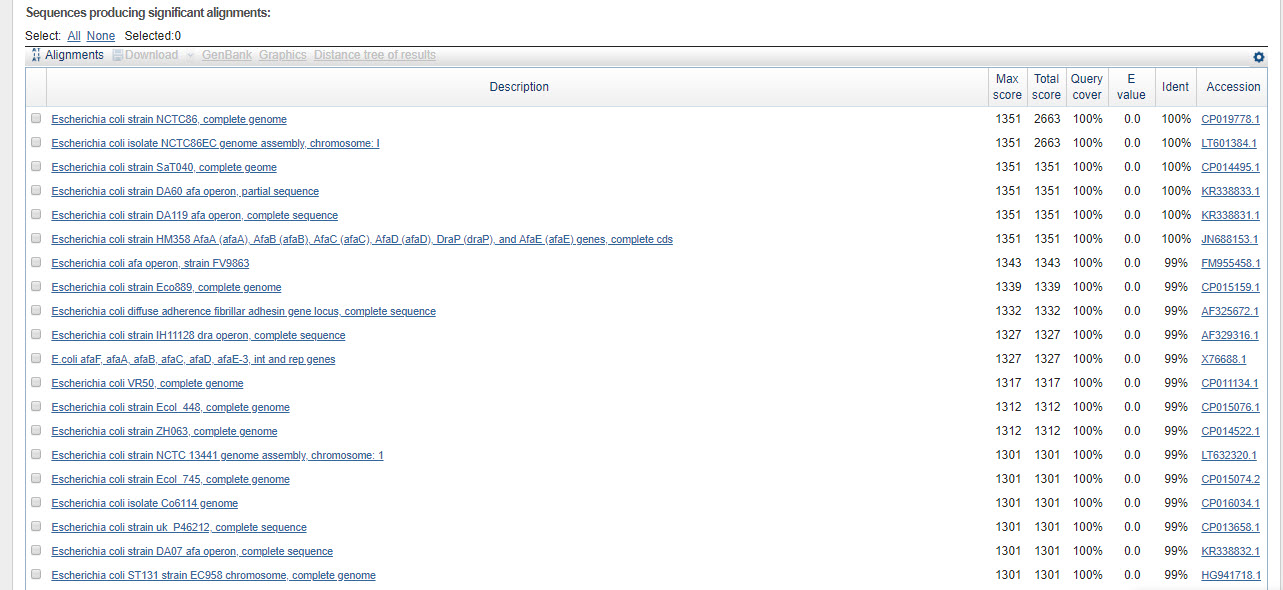


**Results of protein BLAST through http://www.uniprot.org/blast**


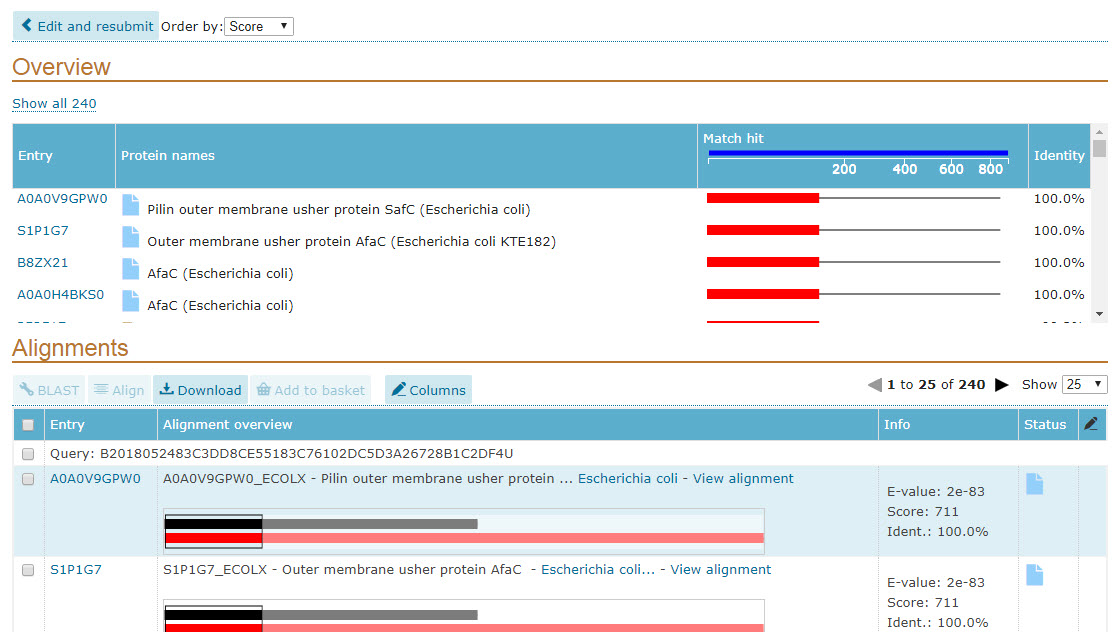

Supplement: Supplementary file 1 — The sequencing results of the afa gene. (DOCX 485 kb) [file 12879_2018_3467_MOESM1_ESM.docx]
